# Pediatric Sarcoma Data Forms a Unique Cluster Measured via the Earth Mover’s Distance

**DOI:** 10.1038/s41598-017-07551-8

**Published:** 2017-08-01

**Authors:** Yongxin Chen, Filemon Dela Cruz, Romeil Sandhu, Andrew L. Kung, Prabhjot Mundi, Joseph O. Deasy, Allen Tannenbaum

**Affiliations:** 1 Memorial Sloan Kettering Cancer Center, Department of Medical Physics, New York, 10064 USA; 2 Memorial Sloan Kettering Cancer Center, Department of Pediatrics, New York, 10064 USA; 3 Stony Brook University, Department of Biomedical Informatics, Stony Brook, 11794 USA; 4 Columbia University, Department of Medical Oncology, New York, 10032 USA; 5 Stony Brook University, Departments of Computer Science and Applied Mathematics & Statistics, Stony Brook, 11794 USA

## Abstract

In this note, we combined pediatric sarcoma data from Columbia University with adult sarcoma data collected from TCGA, in order to see if one can automatically discern a unique pediatric cluster in the combined data set. Using a novel clustering pipeline based on optimal transport theory, this turned out to be the case. The overall methodology may find uses for the classification of data from other biological networking problems.

## Introduction

The present note describes a novel method for data clustering applied to the classification of pediatric sarcoma data. Namely, in this work, we combined two data sets: the first consisting of the gene expression of predominantly pediatric sarcoma patients, and the second consisting of the gene expression of adult sarcoma patients taken from the The Cancer Genome Atlas (TCGA) database. We then wanted to see if one could discern some quantifiable difference between the pediatric and adult cases.

Accordingly, we applied a method based on the *L*_1_ Earth Mover’s Distance (EMD) to the data; see Sections and below for all the details. In what follows EMD will always refer to the *L*_1_ version of the Earth Mover’s Distance. Briefly, the proposed pipeline constructs a weighted graph based on the network topology inferred from the Human Protein Reference Database (HPRD), and then treating the graph as a Markov chain, constructs the invariant (stationary) measure, computes the pairwise distances via EMD among all the networks, and then represents the resulting distance matrix as a heat map. (We note that a *heat map* is a graphical representation of data in which the individual values contained in a matrix are represented as colors.) Other than an outlier (see Section below), our method was able to segregate the pediatric cases from the adult cases, i.e., we found two rather distinct clusters.

We should note that ideas based on the *L*_1_ Earth Mover’s Distance (also known as the Wasserstein 1-metric^1–3^) have already been applied in studying various properties of cancer networks. In particular, the Wasserstein 1-metric leads to a notion of curvature^4^ that turns out to be positively correlated with network robustness^5–7^. This geometric network approach to studying cancer, led to some work indicating that cancer networks are more functionally robust than their normal counterparts^6, 7^.

The EMD (and more generally optimal mass transport theory) is very natural for studying the properties of various weighted graphs modeling biological networks, since it gives a natural metric between probability distributions. Its use has become very widespread in recent years being employed for problems in communications, finance, engineering, and biology^1–3, 8^. This work continues this line of research, by using the distance to cluster biological data.

Finally, we believe that the overall pipeline can be more generally applied in clustering many different types of network data (represented as a weighted graph). We note that we associate the invariant measure to each individual network in the class of data to be classified, and then apply the EMD. This is a distinct advantage since no preprocessing is necessary, other than normalizing the weighted graphs to ensure that they define a Markov process^9^.

## Results

### Data

The gene expression data sets used in the present work, consist of two parts. The first part includes the gene expression of 27 patients diagnosed with pediatric-associated sarcoma and treated at Columbia University Medical Center (CUMC). Informed and signed consent for clinical and research sequencing was obtained in the context of the pediatric precision medicine program (PIPseq) established at CUMC and under the CUMC Institutional Review Board (IRB)-approved protocol AAAN8404^10^. The second part was downloaded from The Cancer Genome Atlas (TCGA) database, covering the gene expression data of 265 adult patients. We have one sample per patient for both of them, so 292 samples in total. The data sets were normalized utilizing one of the standard methods for treating RNA-Seq counts data via the variance-stabilizing transformation (VST) in the DESeq2 package for R^11^. This normalization was done amongst all of the 292 sarcoma samples.

The network topology (graph adjacency matrix) was constructed using interaction information from the Human Protein Reference Database (HPRD)^12^. Specifically, we took the intersection of the genes that appear in both HPRD data and the gene expression data, and then kept the largest connected component. After discarding the redundant genes, we arrived at a gene regulatory network with 8844 nodes (genes) and 34926 edges (interactions). The average and median degrees are 7.9 and 4, respectively.

### Weighted graph and invariant measure

We constructed a weighted graph for each sample using the mass action principle^13^. In particular, for given gene expression {*x*_*i*_ > 0|1 ≤ *i* ≤ *n*} the weight *p*_*ij*_ on the edge (*i*, *j*) is defined as

1 $${p}_{ij}=\frac{{x}_{j}}{{\sum }_{k\in N(i)}{x}_{k}}$$

for any *j*∈*N*(*i*). Here *n* = 8844 is the number of nodes and *N*(*i*) denotes the set of neighbors of the node *i*. Note by construction the matrix $P={[{p}_{ij}]}_{i,j=1}^{n}$ is a stochastic matrix and satisfies that *p*_*ij*_ = 0 if the edge (*i*, *j*) doesn’t exist. Biologically speaking, mass action is similar to well established methods of differential gene co-expression^14^ utilized to develop specific profile for metastatic states^13^. Here, similar to differential co-expression or correlation for analyzing co-regulation patterns in cellular pathways, mass action is based on the assumption that intensity of the interaction between two interactive genes is likely to be larger if both of them have higher expression level. For example, often in drug studies^15, 16^, one studies co-regulation patterns via the differential expression of genes that are induced through a knockdown of separate gene (e.g., PI3K inhibition of BYL719 induces expression of estrogen receptor function in breast cancer^15^). Here, the same underlying principles are used when employing mass action with the added advantage that one can construct patient/sample specific networks without the usage of multiple samples needed for correlation.

The stochastic matrix *P* defines a Markov chain^9^ on the gene regulatory network. Different properties such as entropy and curvature have been considered for this object to study robustness of cancer network^6, 7, 17^. Here we consider the invariant measure (stationary distribution) of this Markov chain. The Markov chain describes the information flow between genes. When the underlying network is connected, the system will eventually reach an equilibrium and this equilibrium is described by the invariant measure. Mathematically, it is a probability vector *π* satisfying

2 $$\pi P=\pi .$$

Thus *π* is a left eigenvector of *P* with non-negative entries that sum to 1. The value *π*_*i*_ at node *i* reflects the portion of contribution of that node to the entire network. In other words, the invariant measure *π* is a centrality measure of the significance of different genes.

In general, to obtain the invariant measure, one needs to solve the linear equation (2). However, for the specific stochastic matrix in (1), *π* has the explicit structure

3 $${\pi }_{i}=\frac{1}{Z}{x}_{i}\sum _{j\in N(i)}\,{x}_{j}$$

where *Z* is a normalization factor (partition function) forcing *π* to be a probability vector.

The expression (3) is very interesting. Note that the value of *π*_*i*_ at node *i* reflects the significance of gene *i* in the gene regulatory network. It consists of two components: the gene expression level *x*_*i*_ of gene *i* and the total gene expression of its neighbors ${\sum }_{j\in N(i)}\,{x}_{j}$. *In other words*, *the invariant measure captures the key property that a gene is important if its expression level is high and it interacts with many other genes*.

### Optimal transport on graphs

Consider a connected undirected graph ${\mathscr{G}}=({\mathscr{V}}, {\mathcal E} )$ with *n* nodes in ${\mathscr{V}}$ and *m* edges in ${\mathcal E}$. Given two densities ${\rho }^{0},\,{\rho }^{1}\in {{\mathbb{R}}}^{n}$ on the graph, the original formulation of the optimal transport problem seeks a joint distribution $\mu \in {{\mathbb{R}}}^{n\times n}$ of *ρ* ^0^ and *ρ* ^1^ minimizing the total cost $\sum \,{c}_{ij}{\mu }_{ij}$, that is,

4 $${W}_{1}({\rho }^{0},{\rho }^{1})=\mathop{{\rm{\min }}}\limits_{\mu }\{\sum _{i,j=1}^{n}\,{c}_{ij}{\mu }_{ij}|\sum _{k}\,{\mu }_{ik}={\rho }_{i}^{0},\,\sum _{k}\,{\mu }_{kj}={\rho }_{j}^{1},\,\forall i,j\}.$$

Here *c*_*ij*_ is the cost of moving unit mass from node *i* to node *j* and is taken to be the minimum of the number of steps to go from *i* to *j*, namely, *c* is the ground metric on the graph. For example, if the edge (*i*, *j*) exists, then *c*_*ij*_ = 1. The minimum of this optimization problem defines a metric *W*_1_ (the Earth Mover’s Distance) on the space probability densities on ${\mathscr{G}}$. An alternative formulation (see Methods) is defined on the fluxes $u\in {{\mathbb{R}}}^{m}$ given on the edges. Let $D\in {{\mathbb{R}}}^{n\times m}$ be the oriented incidence matrix of ${\mathscr{G}}$, then

5 $${W}_{1}({\rho }^{0},{\rho }^{1})=\mathop{{\rm{\min }}}\limits_{u}\{\sum _{i=1}^{m}\,|{u}_{i}||{\rho }^{0}-{\rho }^{1}-Du=0\}.$$

Note that the incidence matrix $D=[{d}_{ik}]\in {{\mathbb{R}}}^{n\times m}$ is defined by associating an orientation to each edge *e*_*k*_ = (*i*, *j*) = (*j*, *i*) of the graph: one of the nodes *i*, *j* is defined to be the head and the other the tail, and then we set *d*_*ik*_ = +1(−1) if *i* is the head (tail) of *e*_*k*_ and 0 otherwise. Compared to (4), which has *n* ^2^ variables, the above formulation has only *m* variables. It may greatly reduce the computational load when the graph ${\mathscr{G}}$ is sparse, i.e., $m\ll {n}^{2}$. This is the case in our data sets, where *n* = 8844 and *m* = 34926. In implementation, we used the standard convex optimization package CVX^18^ written in Matlab, in order to numerically solve (5). We should also note that there is some very nice recent work on the fast computation of the Earth Mover’s Distance^19^ based on (5).

### Clustering of sarcoma data

**Figure 1:**
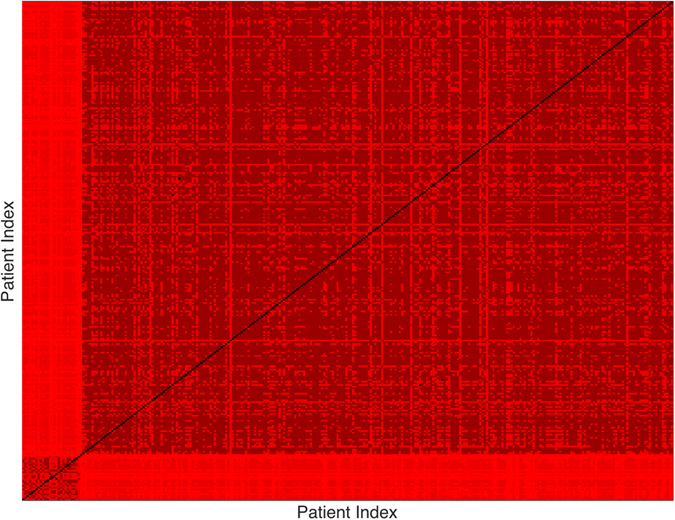
Heat Map Showing Pediatric Cluster.

We define a distance function between different gene expression data sets using optimal transport theory on graphs. More specifically, we define the distance between two gene expression data sets to be the *W*_1_ optimal mass transport distance between the two invariant measures induced by the gene expressions as in (3). This distance *W*_1_ can be computed through convex programming^1, 8^. We computed the *W*_1_ distances between each pair of all the 292 samples (27 pediatric sarcoma and 265 adult cancer). The heat map of the resulting distance matrix is as shown in Fig. 1. The samples clearly split into two clusters; one cluster for the 27 pediatric sarcoma samples and one cluster for the 265 adult cancer patients.

**Figure 2:**
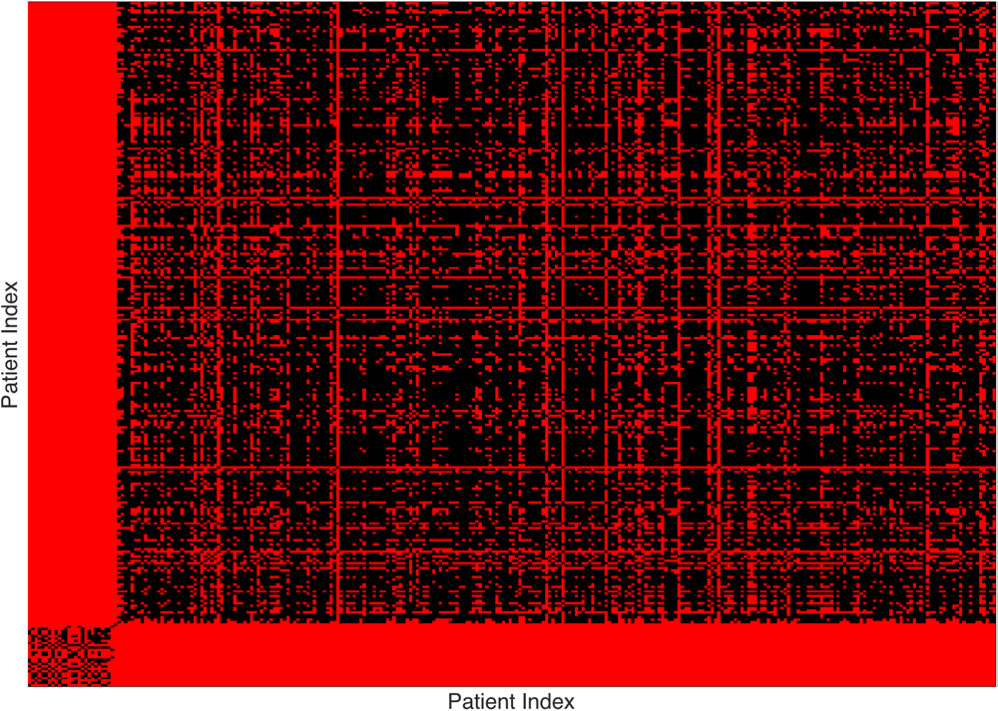
Heat Map Showing Pediatric Cluster with Threshold Value 0.075.

**Figure 3:**
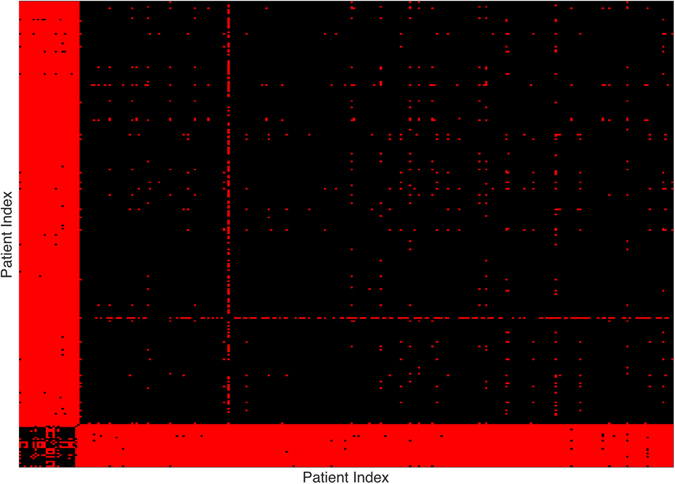
Heat Map Showing Pediatric Cluster with Threshold Value 0.1.

**Figure 4:**
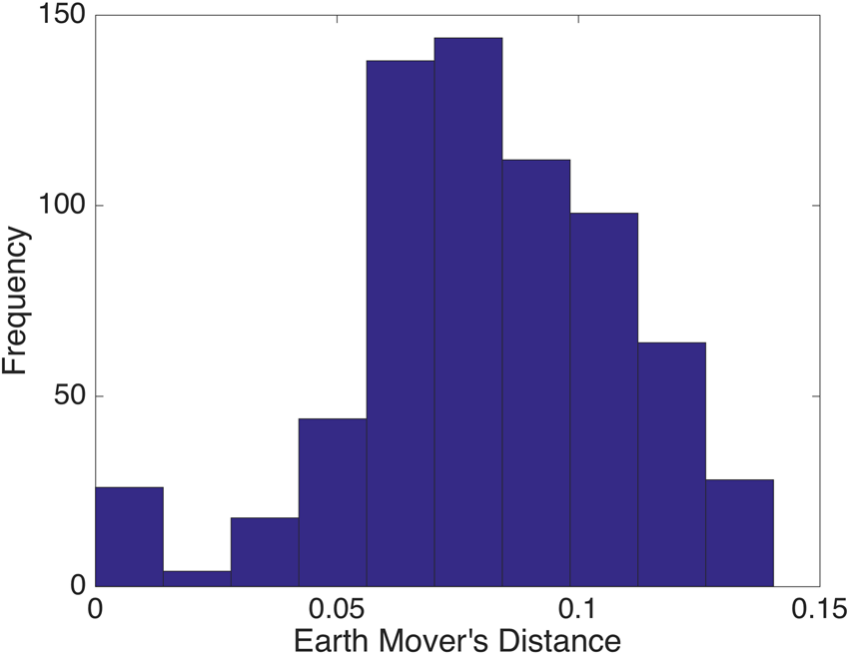
3D Plot Showing Pediatric Cluster.

**Figure 5:**
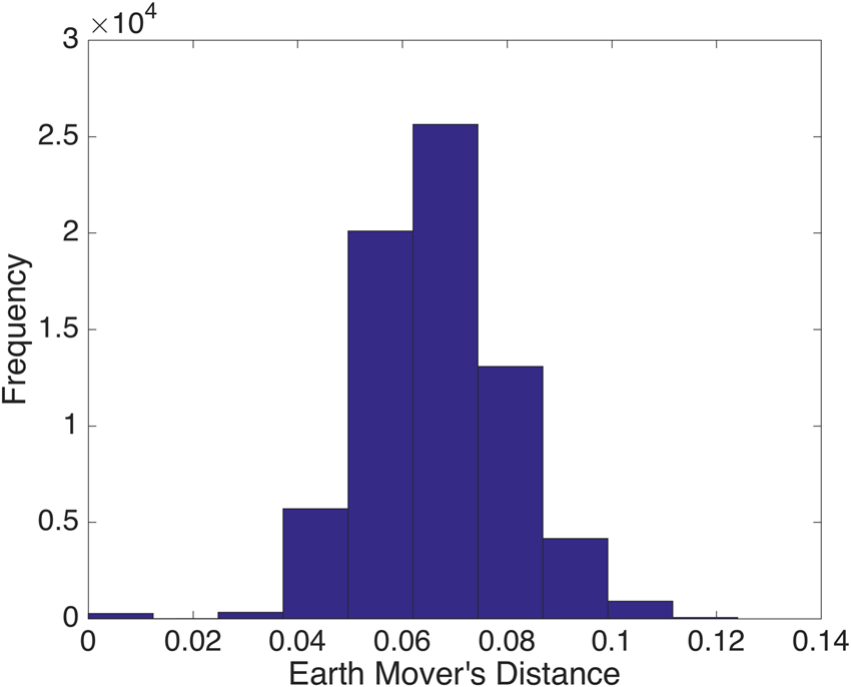
Distances within the Pediatric Cluster.

**Figure 6:**
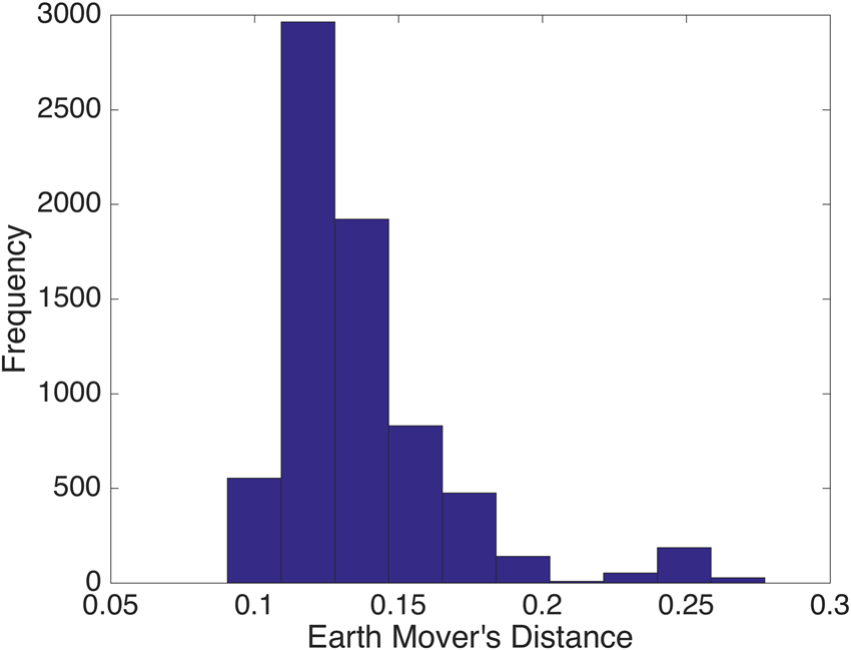
Distances within the Adult Cluster.

**Figure 7:**
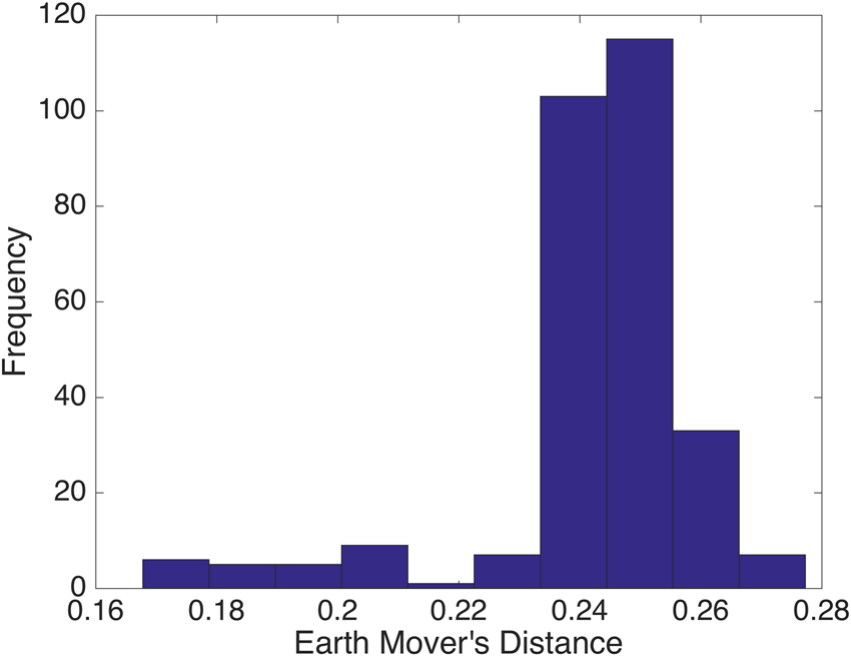
Distances between Pediatric Cluster and Adult Cluster.

**Figure 8:**
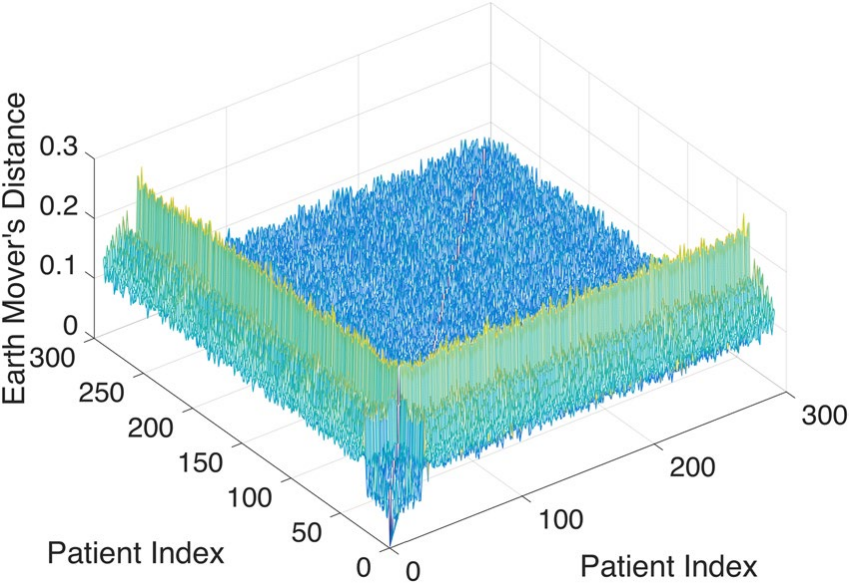
Distances between the Outlier (PIP13-81192) and the other Samples.

To visualize more clearly the two clusters, we truncate the distances using some threshold: set the value to be zero if the distance is less than the given threshold and one otherwise. The results with threshold value 0.075 and 0.1 are depicted in Figs 2 and 3, respectively. Note that there is a small gap between these two clusters, which indicates that the last sample in the pediatric sarcoma is an outlier. Figure 4 is a 3D plot of the distance matrix, from which we can see an obvious difference that distinguishes this outlier from the rest of the sarcoma samples. The clusters and the outlier can be also seen based on the histograms. Figures 5, 6 and 7 are the histograms of the distances within the pediatric sarcomas, within the adult sarcomas, and between these two age groups, respectively. Apparently the distances within the two groups (pediatric, adult) are smaller than the distances between them. In particular, the average distances within the two groups are 0.0891, 0.0665 while the average distance between them is 0.1366. The distance between the outlier and the other samples is shown in Fig. 8, with mean value 0.2424, which is significantly larger than the average. See our discussion in the next section for further analysis of these results.

## Discussion

Sarcomas represent a heterogeneous group of malignant solid tumors of connective tissue. Sarcomas comprise approximately 1.5% of all malignant tumors diagnosed in adults and over 7% of cancers in children^20^. Although the diversity of sarcoma subtypes can be encountered across the age spectrum, there exists a pattern of sarcoma subtypes that significantly distributes between adults and children. For example, osteosarcoma and Ewing sarcoma (malignant bone tumors) are predominant in children and early adults, whereas undifferentiated pleomorphic sarcoma (previously called malignant fibrous histiocytoma), liposarcoma and leiomyosarcoma are extremely rare in children^21, 22^.

In addition to the observation that particular sarcoma subtypes predominate in either childhood or in adulthood, there are also differences in the clinical outcomes of adult and childhood sarcoma patients that extend beyond the differences in treatment regimens between adult and childhood sarcomas^21, 23–25^. With the emergence of next-generation sequencing technologies, we are afforded the opportunity to evaluate the biologic differences between pediatric-associated and adult-associated sarcomas.

In our analysis of 27 sarcoma cases treated at CUMC, only 26 of the 27 original cases would be categorized as a pediatric-associated sarcoma. Interestingly, one case originally included in the pediatric set segregated as an ***outlier***. This case represents a 25 year old female with a history of multiply relapsed, metastatic alveolar soft part sarcoma (ASPS). ASPS is a rare sarcoma subtype comprising 0.2–0.9% of all soft tissue sarcomas^26^. ASPS is extremely rare in childhood, and is more commonly diagnosed in adolescence and young adulthood (15–35 years of age)^20^.

A second adult case included in the pediatric cohort is from a 38 year old male with metastatic synovial sarcoma. In contrast to the previous adult cases of ASPS, this case segregated with the pediatric cohort. Synovial sarcoma is a soft tissue sarcoma with a peak incidence in the 3rd decade of life, and with about 1/3 of cases occurring within the pediatric age range^27^. Synovial sarcoma is more common than ASPS and is the most frequent non-rhabdomyosarcomatous soft tissue sarcoma in adolescents and young adults^28^. Although historical differences in the approach to therapy between pediatric and adult oncologists have existed for the treatment of sarcomas and other tumors, there has been acknowledgement in the adult oncology community of the clinical utility of pediatric-based regimens for the treatment of sarcomas occurring in adulthood^29, 30^. However, despite use of more dose-intense chemotherapeutic approaches to the treatment of sarcomas in adulthood, pediatric-associated sarcomas diagnosed and treated in adulthood continue to have inferior outcomes compared to treatment in childhood^31, 32^.

These observations suggest that there may exist age-dependent differences in the biology of sarcomas. However, it is unclear what the thresholds for age may be that would contribute to differential responses to treatment and clinical outcome as the cutoffs for age and the definition of “adult age” has varied in the literature. The results from this analysis suggest that the sarcoma subtype may supersede, in this instance, the contribution of age to the biologic behavior and genomic signature. So from this classification scheme, it seems that there are indeed biologic differences between sarcoma subtypes that are generally associated with childhood (such as synovial sarcoma) versus those more commonly associated with adulthood (such as ASPS), and provides a rationale for the use of pediatric regimens for the treatment of these diseases regardless of the patient’s age.

Genomic characterization of a larger cohort of pediatric-associated and adult-associated sarcomas will be imperative in specifically clarifying the genomic lesions that result in the clinical differences in behavior of sarcomas across the age spectrum. In any event, we did manage to cluster 26 out of the 27 CUMC cases from the TCGA data using our methodology.

In our Supplemental Information file, we have two other examples. The first illustrates the methodology applied to clustering breast cancer data (triple negative and normal). The second using synthetic “gene expression” networks shows the importance of topology in clustering. EMD has the nice feature of explicitly utilizing the topology of the network under consideration.

**Figure 9:**
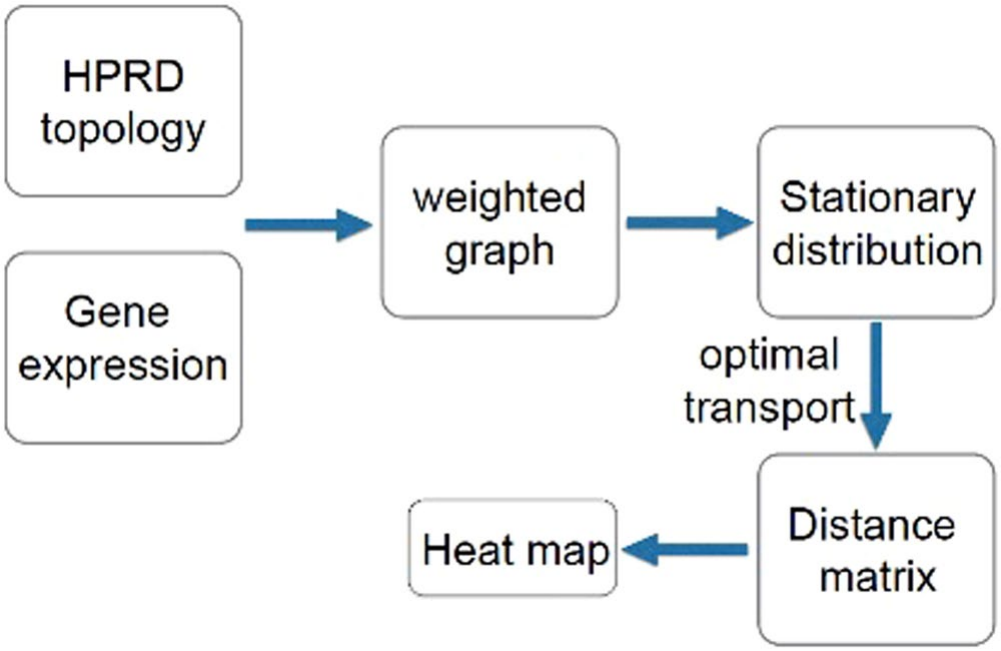
Overall Sketch of Method.

We should finally note that the pipeline sketched in Fig. 9 is quite general and may be quite useful in clustering various biological networks. These typically may be represented as weighted graphs, and thus after suitable normalization as Markov chains for which there exist the corresponding stationary measures. Optimal mass transport theory realized by the Earth Mover’s Distance seems to be an ideal tool for capturing distances among these measures, and thus leads to a natural clustering/classification framework. Several interesting biological graphs as suggested by one of the reviewers could include those based on evolutionary distance between genes, structural similarity in within same fold family, percent of shared functional sites, even predicted, and percent of shared protein domains.

## Methods

### Overall sketch

Figure 9 illustrates the overall pipeline of the clustering methodology described in the previous sections. The basic idea is that once one has defined the network topology (in this case via the Human Protein Reference Database), and the weights connecting the nodes (derived here from the mass action principle), one can use in a straightforward manner an invariant of each network, and then compute the distance matrix defined by the EMD or Wasserstein 1-metric. In the next section, we will review the definition and properties of this central mathematical object underpinning our analysis.

### Earth Mover’s Distance

**Figure 10:**
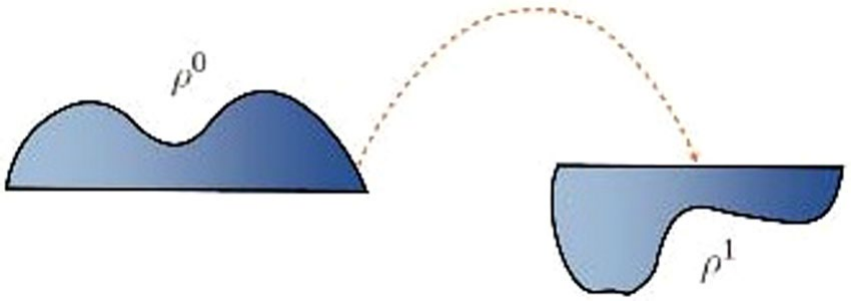
Classical Earth Mover’s Problem: The dashed arrow indicates the transport map between the densities *ρ* ^0^ and *ρ* ^1^.

In this section, we briefly review the mathematics of the Earth’s Mover’s Distance (EMD) from optimal mass transport theory, the key method on which all the previous results were based. The classical Earth Mover’s Distance was formulated by Monge in 1781 to solve the problem of moving a pile of soil to a excavation site with the least amount of work relative to some cost. This is illustrated in Fig. 10. For full details as well as long lists of references, see the monographs^1–3^.

Mathematically, we let *ρ* ^0^ and *ρ* ^1^ denote two probability densities on ${{\mathbb{R}}}^{m}$. This means that ${\rho }^{i}:{{\mathbb{R}}}^{m}\to {\mathbb{R}}$ with *ρ* ^*i*^ ≥ 0 for *i* = 0, 1, such that

$${\int }_{{{\mathbb{R}}}^{m}}\,{\rho }^{0}(x)dx={\int }_{{{\mathbb{R}}}^{m}}\,{\rho }^{1}(x)dx=1.$$

Then the *Earth Movers’ Distance* (also called the *Wasserstein 1*-*metric*, *W*_1_) between them is

6 $${W}_{1}({\rho }^{0},{\rho }^{1})\,:=\mathop{{\rm{\min }}}\limits_{\mu \in {\rm{\Pi }}({\rho }^{0},{\rho }^{1})}\,{\int }_{{{\mathbb{R}}}^{m}\times {{\mathbb{R}}}^{m}}\,\Vert x-y\Vert \mu (dx,dy),$$

where Π(*ρ* ^0^, *ρ* ^1^) denotes the set of couplings between *ρ* ^0^ and *ρ* ^1^. The Wasserstein-1 distance has the dual formulation^8^

7 $${W}_{1}({\rho }^{0},{\rho }^{1})=\mathop{{\rm{\sup }}}\limits_{f}\,\{{\int }_{{{\mathbb{R}}}^{m}}\,f(x)\,({\rho }^{0}(x)-{\rho }^{1}(x))dx|{\Vert f\Vert }_{Lip}\le 1\}.$$

Here

$${\Vert f\Vert }_{Lip}\,:=\mathop{{\rm{\sup }}}\limits_{x\ne y}\,\frac{\Vert \,f(x)-f(y)\Vert }{\Vert x-y\Vert }.$$

Clearly when *f* is differentiable, ${\Vert f\Vert }_{Lip}\le 1$ is equivalent to $\Vert {\nabla }_{x}\,f\Vert \le 1$. So formally, the above can be rewritten as

8 $${W}_{1}({\rho }^{0},{\rho }^{1})=\mathop{{\rm{\sup }}}\limits_{f}\,\{{\int }_{{{\mathbb{R}}}^{m}}\,f(x)\,({\rho }^{0}(x)-{\rho }^{1}(x))dx|\Vert {\nabla }_{x}f\Vert \le 1\}.$$

One can then take the dual once again, i.e., starting from (8), one sees that

9 $${W}_{1}({\rho }^{0},{\rho }^{1})=\mathop{{\rm{\inf }}}\limits_{u}\,\{{\int }_{{{\mathbb{R}}}^{m}}\,\Vert u(x)\Vert dx|{\rho }^{0}-{\rho }^{1}+{\nabla }_{x}\cdot u=0\},$$

of *W*_1_ with flux *u* being the optimization variable.

The above “dual of the dual” method can be applied to transport problems on graphs to show the equivalence between (4) and (5) by replacing the metric $\Vert \cdot \Vert$ by *c* and the divergence operator $\nabla \cdot$ by *D*. In so doing, one gets a tremendous saving in computational burden since equation (4) involves solving systems on the order of the ***square*** of the number of nodes, while equation (5) is of the order of the number of edges. In our specific case, we had 8844 nodes (genes) and 34926 edges (interactions), that is, we save in this manner ${8844}^{2}/34926\sim 2\times {10}^{3}$ in the number of variables treated.

## Electronic supplementary material

Supplementary Information

## References

[1] Rachev, S. & Rüschendorf, L. *Mass Transportation Problems*, *Vol*. *I and II* (Springer-Verlag, 1998).

[2] Villani, C. *Optimal Transport*, *Old and New* (Springer-Verlag, 2008).

[3] Villani, C. *Topics in Optimal Transportation* (American Mathematical Society Publications, 2003).

[4] Ollivier Y (2009). Ricci curvature of Markov chains on metric spaces. Journal Functional Analysis.

[5] Demetrius L, Manke T (2005). Robustness and network evolution entropic principle. Physica A.

[6] Sandhu R (2015). Graph Curvature for Differentiating Cancer Networks. Scientific Reports.

[7] Tannenbaum, A. *et al*. Ricci curvature and robustness of cancer networks. http://arxiv.org/abs/1502.04512 (2015).

[8] Evans, L. C. Partial differential equations and Monge–Kantorovich mass transfer. *Current Developments in Mathematics* 65–126 (1999).

[9] Kemeny, J. & Snell, J. L. *Finite Markov Chains* (Van Nostrand, Princeton 1960).

[10] Oberg JA (2016). Implementation of next generation sequencing into pediatric hematology-oncology practice: moving beyond actionable alterations. Genome Medicine.

[11] Love M, Huber W, Anders S (2014). Moderated estimation of fold change and dispersion for RNA-seq data with DESeq2. Genome Biology.

[12] Peri, S. *et al*. Human protein reference database as a discovery resource for proteomics. *Nucleic Acids Res*. bf 32, D497–D501 (2004). 10.1093/nar/gkh070 PMC308804 14681466

[13] Teschendorff A, Sollich P, Kuehn R (2014). ‘Signalling entropy: A novel network-theoretical framework for systems analysis and interpretation of functional comic data. Methods.

[14] Reznik, E. & Sanders, C. Extensive decoupling of metabolic genes in cancer. *PLOS*—*Computational Biology*. doi:10.1371/journal.pcbi.1004176 (2015). 10.1371/journal.pcbi.1004176 PMC4427321 25961905

[15] Bosch, A. *et al*. PI3K inhibition results in enhanced estrogen receptor function and dependence in hormone receptor-positive breast cancer. *Science Translation Medicine*. bf 7 (2015). 10.1126/scitranslmed.aaa4442 PMC4433148 25877889

[16] Lamhamedi-Cherradi, S. *et al*. IGF-1R and mTOR blockade novel resistance mechanisms and synergistic drug combinations for ewing sarcoma. *Journal of the National Cancer Institute* bf 12, 1–10 (2016). 10.1093/jnci/djw182 PMC5241894 27576731

[17] West, J., Bianconi, G., Severini, S. & Teschendorff, A. Differential network entropy reveals cancer system hallmarks. *Scientific Reports* **2**, doi:10.1038/srep00802 (2012). 10.1038/srep00802 PMC3496163 23150773

[18] Boyd, S. & Vandenberghe, L. *Convex Optimization* (Cambridge University Press, 2004).

[19] Li, W., Ryu, E. K., Osher, S., Yin, W. & Gangbo, W. A parallel method for Earth Mover’s Distance. ftp://ftp.math.ucla.edu/pub/camreport/cam17-12.pdf (2017).

[20] Ferrari A (2011). Soft tissue sarcoma across the age spectrum: a population based study from the surveillance epidemiology and end results database. Pediatric Blood Cancer.

[21] Ferrari A (2005). Adult-type soft tissue sarcomas in pediatric-age patients: experience at the Istituto Nazionale Tumori in Milan. J. Clinical Oncol..

[22] Okcu, M. F. *et al*. Nonrhabdomyosarcomatous soft tissue sarcomas. Pizzo, P. A. & Poplack, D. C. (Eds), *Principles and Practice of Pediatric Oncology* (*5th ed*.), Lippincott Williams & Wilkins, Philadelphia, 1033–1073 (2006).

[23] Baker LH (2005). Medical and pediatric oncology, not adult and pediatric oncology. J Clin Oncol.

[24] Bleyer A (2005). National survival trends of young adults with sarcoma: lack of progress is associated with lack of clinical trial participation. Cancer.

[25] Spunt SL, Pappo AS (1958). Childhood nonrhabdomyosarcoma soft tissue sarcomas are not adult-type tumors. J. Clin. Oncol..

[26] Jaber OI, Kirby PA (2015). Alveolar soft part sarcoma. Arch Pathol Lab Med.

[27] Sultan I (2009). Comparing children and adults with synovial sarcoma in the surveillance, epidemiology, and end results program, 1983 to 2005: an analysis of 1268 patients. Cancer.

[28] Weiss, S. & Goldblum, J. Malignant soft tissue tumors of uncertain type. In: Weiss, S. & Goldblum, J. (eds), *Enzinger and Weiss’s Soft Tissue Tumors*, St Louis, Missouri: CV Mosby, 1483–1571 (2001).

[29] Eilber FC (2007). Chemotherapy is associated with improved survival in adult patients with primary extremity synovial sarcoma. Ann. Surg..

[30] Spurrell EL, Fisher C, Thomas JM, Judson IR (2005). Prognostic factors in advanced synovial sarcoma: an analysis of 104 patients treated at the Royal Marsden Hospital. Annals Oncol..

[31] Ferrari A (2004). Synovial sarcoma: a retrospective analysis of 271 patients of all ages treated at a single institution. Cancer.

[32] Ferrari A (2008). Role of chemotherapy in pediatric nonrhabdomyosarcoma soft-tissue sarcomas. Expert Rev. Anticancer Ther..

