# Supplementary material for: Pediatric Sarcoma Data Forms a Unique Cluster Measured via the Earth Mover’s Distance
Source: Sci Rep. 2017 Aug 1;7:7035. doi: 10.1038/s41598-017-07551-8 (PMC5539155; doi:10.1038/s41598-017-07551-8)
Supplement: Supplementary file 1 — Supplementary Information [file 41598_2017_7551_MOESM1_ESM.doc]

SUPPLEMENTARY INFORMATION FILE

Pediatric Sarcoma Data Forms a Unique Cluster Measured via the Earth Mover's Distance

Yongxin Chen^1^, Filemon Dela Cruz^2^, Romeil Sandhu^3^, Andrew L. Kung^2^, Prabhjot Mundi^4^, Joseph O. Deasy^1^, and Allen Tannenbaum^5*^

^1^ Memorial Sloan Kettering Cancer Center, Department of Medical Physics, New York City, NY 10064

^2^ Memorial Sloan Kettering Cancer Center, Department of Pediatrics, New York City, NY 10064

^3^  Department of Biomedical Informatics, Stony Brook University, Stony Brook, NY 11794

^4^  Department of Medical Oncology, Columbia University, New York City, NY 10032

^5^  Departments of Computer Science and Applied Mathematics & Statistics, Stony Brook University, Stony Brook, NY 11794

*corresponding author: allen.tannenbaum@stonybrook.edu

SUPPLEMENTARY DATA

In addition to the sarcoma data set, we opted to supplement our analysis with publically available breast cancer data set available online (GSE454827) (https://www.ncbi.nlm.nih.gov/geo/). In this preliminary study, we are interested in quantifying differences between triple negative (TN) breast cancer and the normal counterpart. Here, due to the metastatic nature and poor survival rates of TN patients, it should be straightforward to see clear delineations in such populations. That is, this serves as a proof of concept for the proposed method. Specifically, the data used consisted of total samples: 52; normal samples: 11; cancer (TN) samples: 41. To construct the underlying protein-to-protein interaction network (PIN), we utilized HPRD database. The total number of genes (nodes) and interactions (edges) were 7044 and 28653, respectively. The resulting weights of each sample was then computed through mass action. Figure S1 is the supplemental figure associated with these results.

SUPPLEMENTARY FIGURE


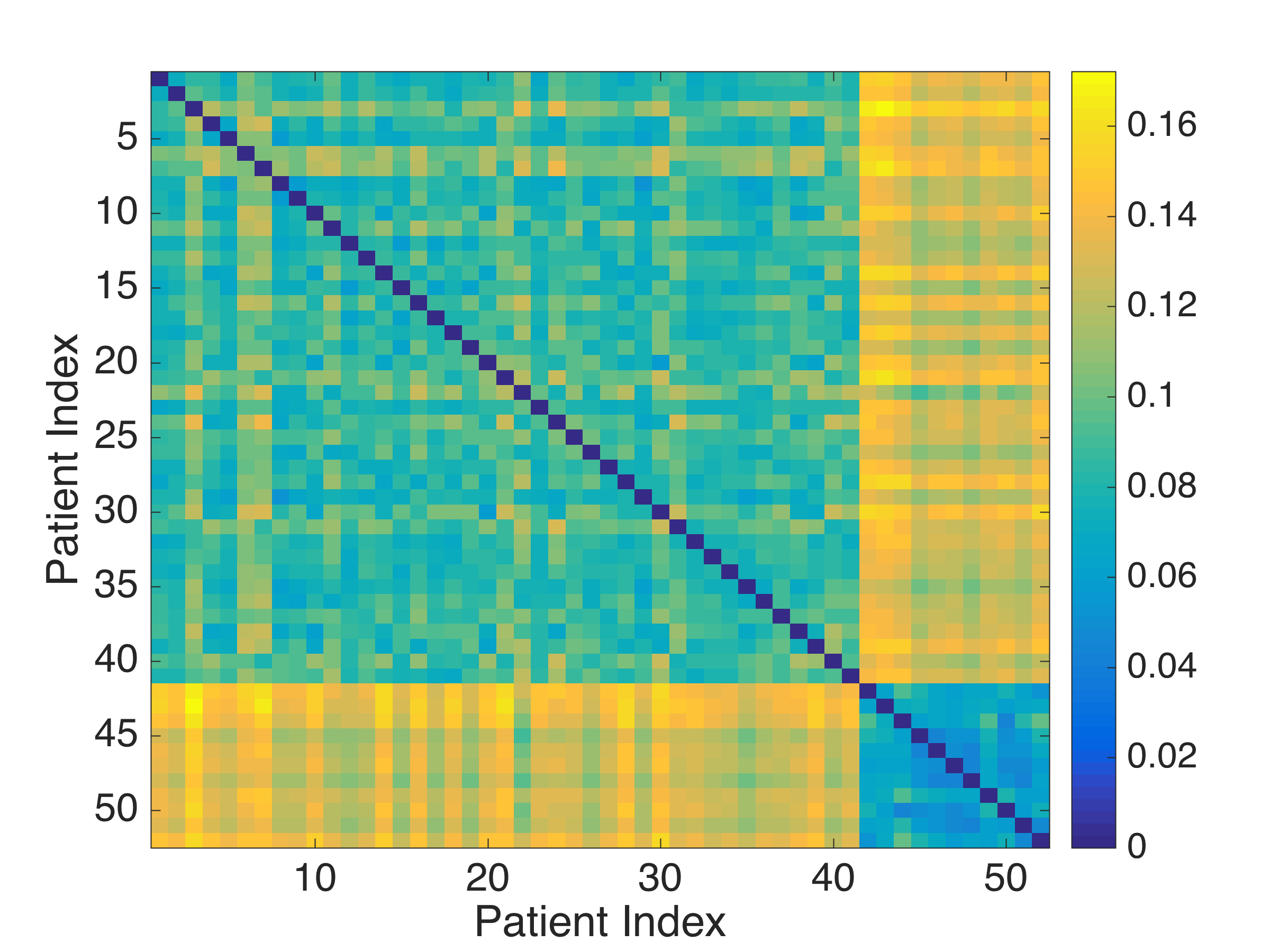


**Supp. Fig. 1:** Heat map of the Earth Mover’s distance between each pair of the 51 samples. The upper left block corresponds to TN breast cancer and the lower right block corresponds to normal samples.

The second example explicitly illustrates our claim that topology may play an essential role in distinguishing samples based on the consideration of the ``gene expressions’’ on two synthetic networks. More specifically, we consider 60 samples of gene expressions: 30 samples for class A and 30 for class B. We generate two synthetic networks: the first one is a scale-free network and the second one is an Erdos-Renyi (ER) random network. Each of the scale-free networks employs the same underlying scale-free graph, and similarly for the ER case. It is believed that biological networks are scale-free networks. The Wasserstein distances between the 60 samples on these two different networks are shown in Figure S2 and Figure S3, respectively. Even though the gene expressions are the same, the distances are clearly very different. While we can see a clean separation between the two classes of samples on a scale-free network, this is not the case on an Erdos-Renyi random network. In comparison, we also display the standard Euclidean distances between the samples in Figure S4. Again, we cannot see any separation between class A and class B.


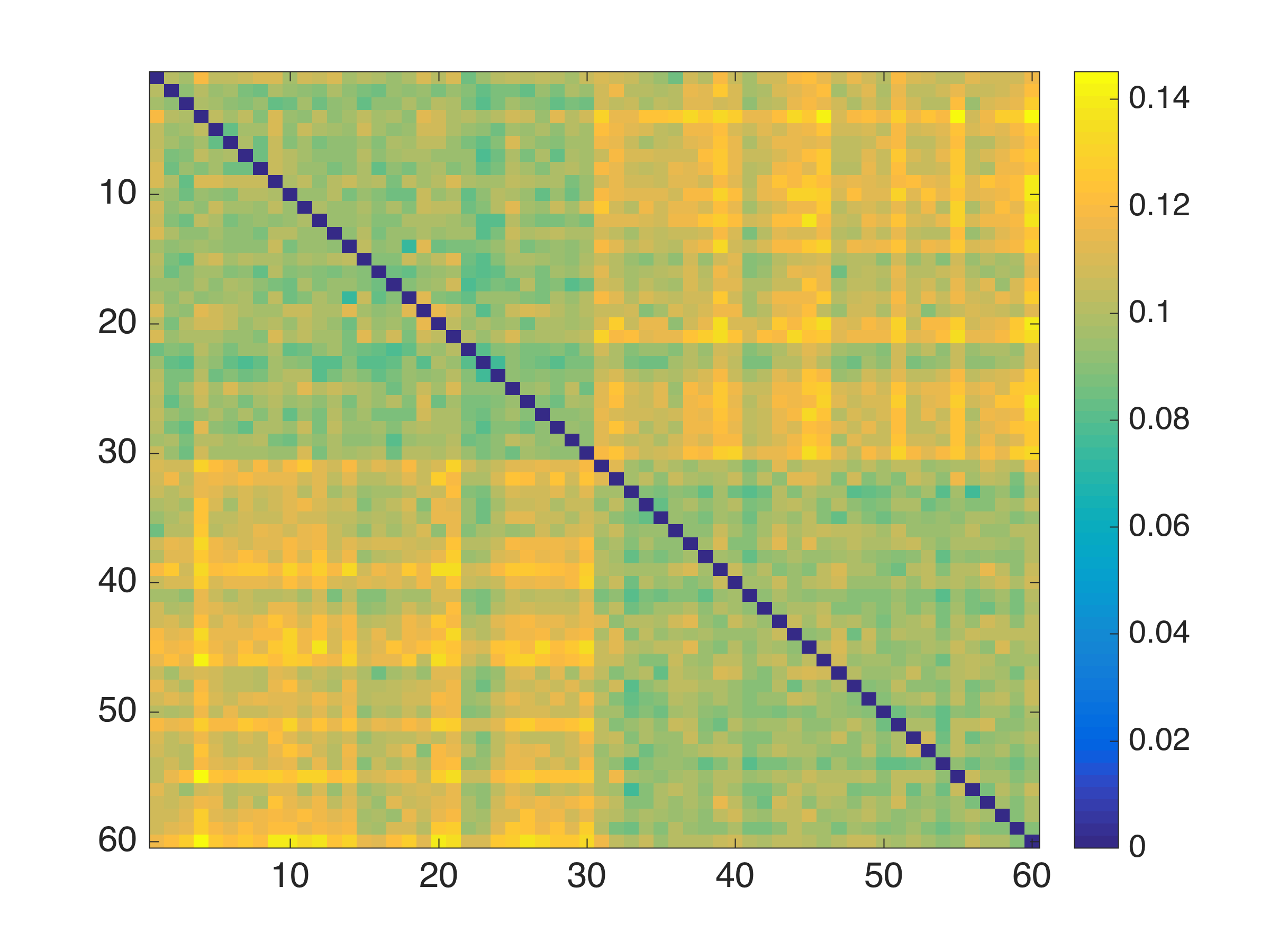


**Supp. Fig. 2:** Heat map of the Earth Mover’s distance between each pair of the 60 samples on a scale free network. The upper left block corresponds to class A and the lower right block corresponds to class B.


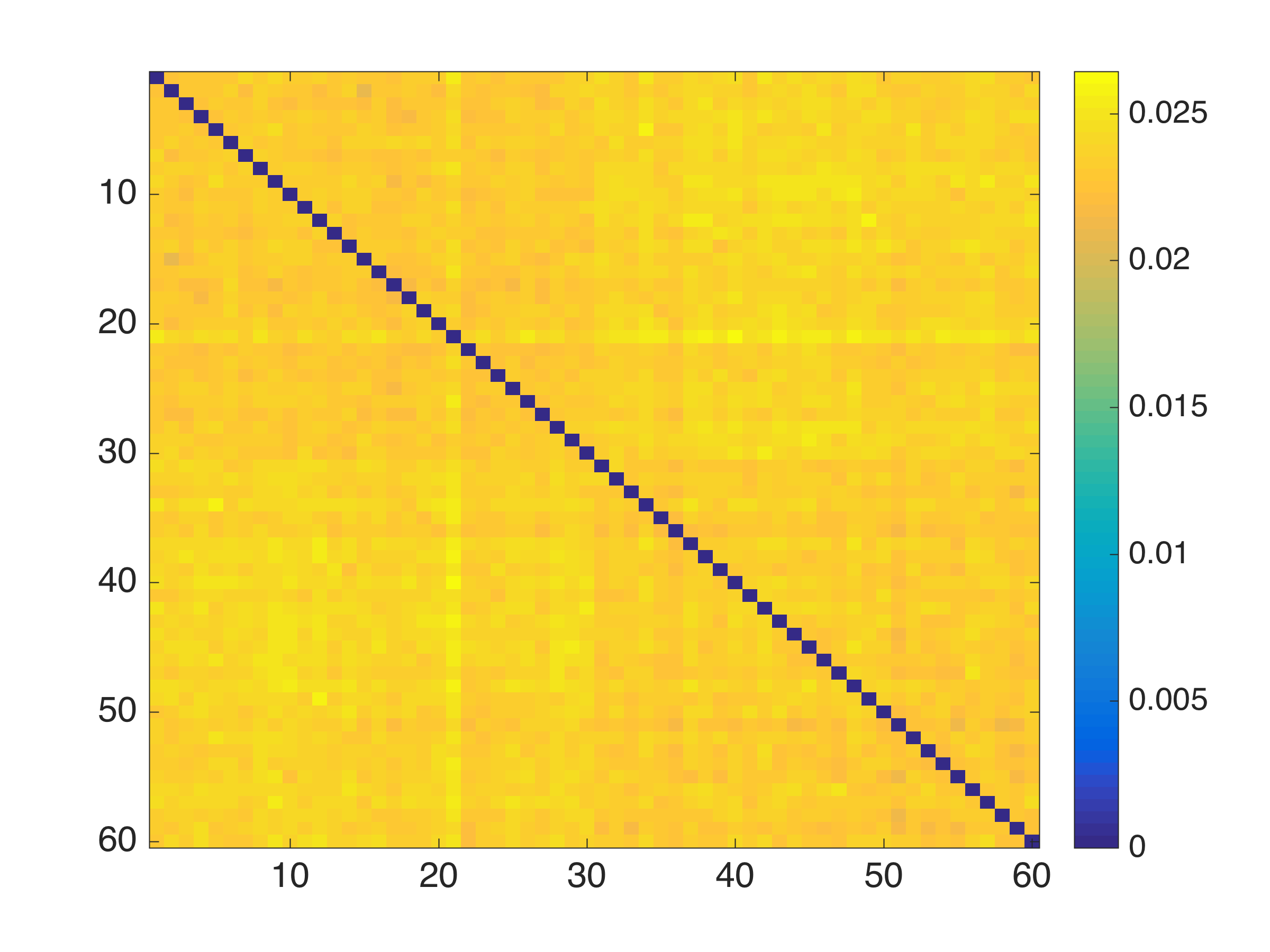


**Supp. Fig. 3:** Heat map of the Earth Mover’s distance between each pair of the 60 samples on an Erdos-Renyi network.


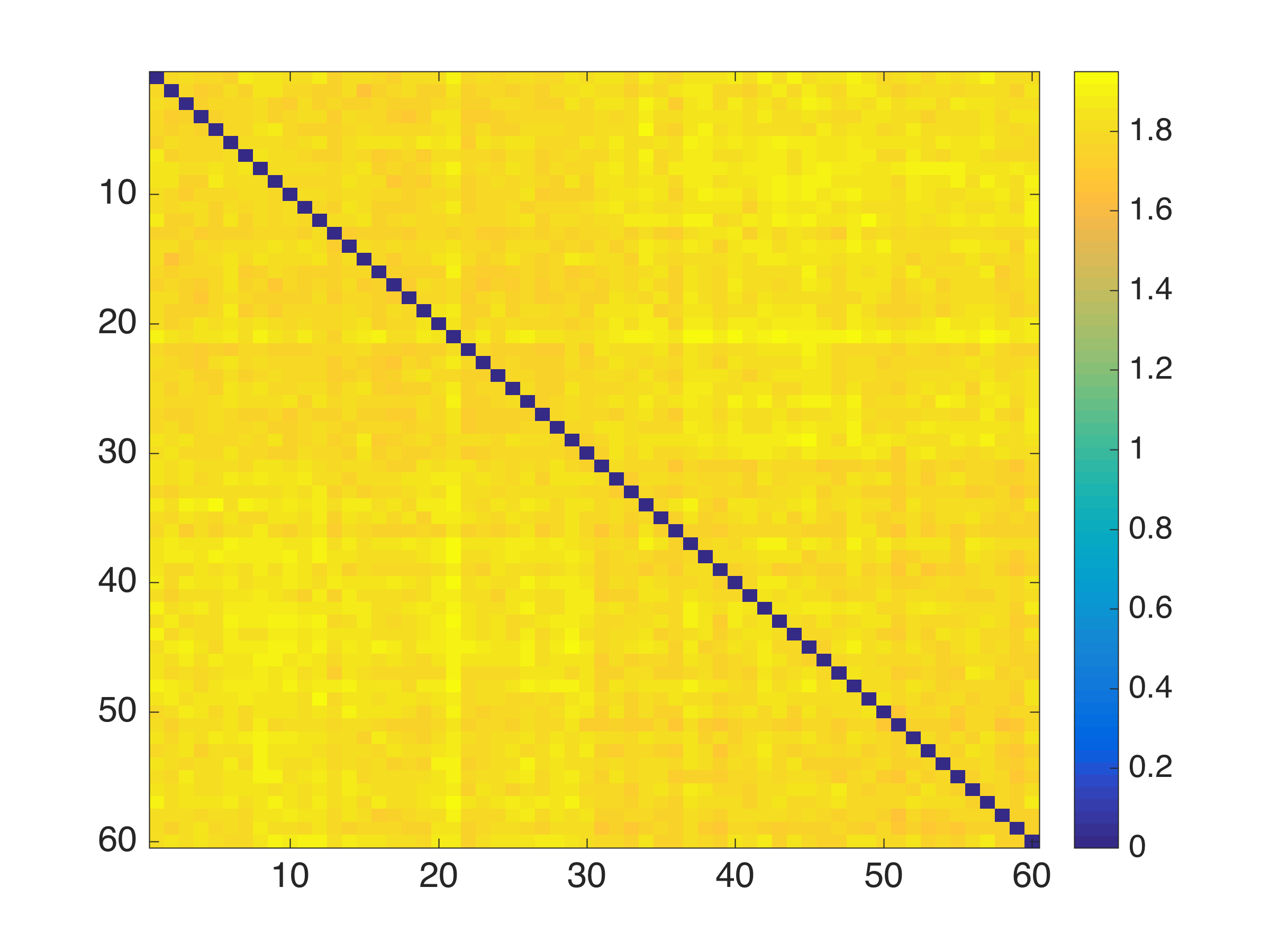


**Supp. Fig. 4:** Heat map of the Euclidean distance between each pair of the 60 samples.
